# Dual Reversible Coumarin Inhibitors Mutually Bound to Monoamine Oxidase B and Acetylcholinesterase Crystal Structures

**DOI:** 10.1021/acsmedchemlett.2c00001

**Published:** 2022-02-18

**Authors:** Fredrik Ekström, Andrea Gottinger, Nina Forsgren, Marco Catto, Luca G. Iacovino, Leonardo Pisani, Claudia Binda

**Affiliations:** ‡ Swedish Defence Research Agency, CBRN Defence and Security, Umeå 901 82, Sweden; § Department of Biology and Biotechnology, University of Pavia, 27100 Pavia, Italy; ∥ Department of Pharmacy-Pharmaceutical Sciences, University of Bari “Aldo Moro”, via E. Orabona 4, 70125, Bari, Italy

**Keywords:** Monoamine oxidase, Acetylcholinesterase, Multitarget inhibitor, Tight-binding, Alzheimer’s disease

## Abstract

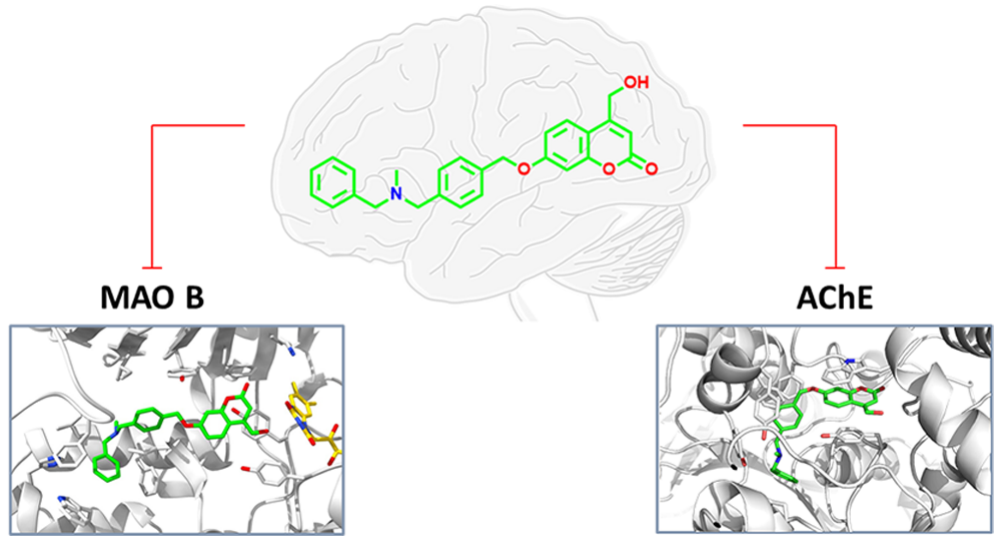

Multitarget directed ligands (MTDLs) represent a promising frontier in tackling the complexity of multifactorial pathologies. The synergistic inhibition of monoamine oxidase B (MAO B) and acetylcholinesterase (AChE) is believed to provide a potentiated effect in the treatment of Alzheimer’s disease. Among previously reported micromolar or sub-micromolar coumarin-bearing dual inhibitors, compound **1** returned a tight-binding inhibition of MAO B (*K*_*i*_ = 4.5 μM) and a +5.5 °C increase in the enzyme *T*_m_ value. Indeed, the X-ray crystal structure revealed that binding of **1** produces unforeseen conformational changes at the MAO B entrance cavity. Interestingly, **1** showed great shape complementarity with the AChE enzymatic gorge, being deeply buried from the catalytic anionic subsite (CAS) to the peripheral anionic subsite (PAS) and causing significant structural changes in the active site. These findings provide structural templates for further development of dual MAO B and AChE inhibitors.

Polypharmacological protocols that are based on drug cocktails have entered the clinical practice to treat complex pathological conditions such as cancer^1^ and cardiovascular diseases,^2^ whose onset and progression build on multiple molecular mechanisms. A multifaceted landscape of cellular processes is shared by many neurodegenerative diseases (NDs), such as oxidative stress, biometals imbalance, protein misfolding, and aggregation that have been observed in brains affected by Alzheimer’s (AD) or Parkinson’s disease (PD).^3,4^ Unfortunately, despite huge efforts engaging several potential drug targets, no curative treatment has been made available thus far.^5,6^ In the last two decades, medicinal chemistry programs focused on NDs shifted from the well-established “one drug–one target” paradigm to the attractive and promising multitarget directed ligands (MTDLs) strategy.^7,8^ Multipotent drugs, ideally not promiscuous^9^ and intentionally designed to modulate at least two relevant targets,^10,11^ may benefit from additive and/or synergistic activities. The choice of networked targets is a crucial step to be addressed by phenotypic screening, system biology, and *in silico* technologies. A web-based survey of scientific literature revealed the impact of multitargeting drugs as a topic growing at exponential rate in more recent years.

Within the plethora of unpaired neuronal mechanisms proposed as druggable targets for AD,^12^ the discovery of ladostigil^13^ signed a milestone in studying the dual-targeting inhibition of acetylcholinesterase (AChE) and monoamine oxidases (MAOs). This dual-acting prototype inhibitor was endowed with a covalent mechanism of action (pseudoirreversible for AChE, irreversible for MAO). Two decades after the launch of ladostigil, AChE/MAO is still one of the most relevant target combinations, addressed with both covalent and reversible inhibitors proposed to combat AD.

AChE regulates cholinergic transmission at the synaptic levels by hydrolyzing the neurotransmitter acetylcholine (ACh). Actually, AD treatment mainly involves the administration of orally active AChE inhibitors exerting merely palliative, symptomatic relief.^14^ Monoamine oxidases A and B (MAO A and B) are enzymes cleaving the C_α_–N bond of several arylalkylamines (including xenobiotics and neurotransmitters such as dopamine) upon a FAD-dependent oxidative deamination reaction.^15^ MAO A and B are validated drug targets for depression and for PD, respectively, whereas, more recently, a renewed interest in these enzymes arose from their role in cardiac senescence^16^ and other age-related disorders, as for AD.^17−19^

Over the years, much attention has been given to both AChE and MAO B in the context of dual-target inhibitors for AD treatment, as inferred by the number of X-ray crystallographic structures retrieved within the Protein Data Bank (PDB) comprising apo states and binary complexes (from different species in the case of AChE) with covalent or reversible inhibitors. However, few X-ray structures described the binding mode of multimodal compounds targeting MAO B (covalently) or AChE (reversibly), whereas to our knowledge this is the first study reporting the binding poses of two dual reversible coumarin-based AChE-MAO B inhibitors (**1** and **2**, Figure 1)^20,21^ on the crystal structures of both enzymes.

**Figure 1:**
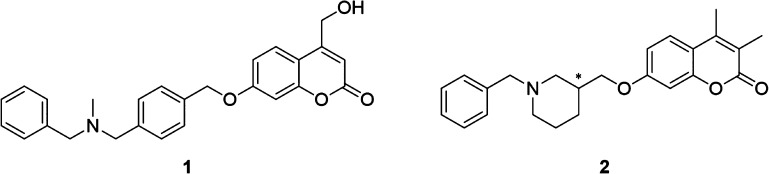
Chemical structure of 7-[(4-{[benzyl(methyl)amino]methyl}benzyl)oxy]-4-(hydroxymethyl)-2*H*-chromen-2-one (**1**, as hydrochloride) and 7-[(1-benzylpiperidin-3-yl)methoxy]-3,4-dimethyl-2*H*-chromen-2-one (**2**) in either its racemic mixture (hydrochloride) or as enantiopure forms (free bases).

Coumarin is a nature-inspired, privileged scaffold that has been differently decorated in the search of single- and multitargeting inhibitors of AChE and MAO by several research groups.^22−24^ Both **1** and **2** (racemic mixture or enantiopure samples) were previously identified as dual-targeting hits by some of us. As for coumarin **1**, a design-in approach inspired the introduction of the pendant *N*-methylbenzylamine moiety to the 7-benzyloxycoumarin framework (known as MAO B hitter) with the aim of improving AChE inhibition.^20^ Compound **2** was discovered by hybridizing the coumarin nucleus with the donepezil-based *N*-benzylpiperidine template.^21^ For all inhibitors, by using commercial sources of MAO and AChE enzymes, the IC_50_ values were in the micromolar or sub-micromolar range (Table 1). This observation prompted us to investigate in more detail the inhibition mechanism and binding mode of **1** and **2** relative to their dual enzyme targets using purified recombinant forms of human MAO B and mouse AChE. The latter shares 88% sequence identity with the human enzyme.^25^

**Table 1:** Inhibition of MAO B and AChE Enzymes by Compounds **1** and **2** (*K*_i_ and IC_50_ Values, Both Expressed as μM)

	MAO B		AChE	
	human		human	mouse
	*K*_*i*_^a^	IC_50_^b^^,^^c^	IC_50_^d^	IC_50_^e^
**1**	4.5 ± 0.2^f^	0.010 ± 0.002	0.12 ± 0.01^c^	0.40 ± 0.03
**(+)-2**	0.093 ± 0.015	0.023 ± 0.003	1.6 ± 0.1	1.5 ± 0.1
(−)-**2**	0.19 ± 0.02	0.19 ± 0.08	1.3 ± 0.2	0.70 ± 0.05
**(±)-2**	0.13 ± 0.02	0.030 ± 0.005	1.4 ± 0.3	n.d.

a Spectrophotometric experiments through HRP-coupled assay applied on purified recombinant enzyme.

b Fluorescence method (kynuramine) applied on recombinant enzyme from commercial sources.

c IC_50_ values have been already reported in the literature.^1,2^

d Spectrophotometric Ellman’s method applied on recombinant enzyme from commercial sources.

e Spectrophotometric Ellman’s method applied on purified recombinant enzyme.

f Tight-binding *K*_i_ determined by Morrison equation (see text).

In our contribution to the field, we reported the crystal structure complexes with human MAO B of single-targeting compounds.^26^ Therefore, we first undertook a thorough kinetic analysis of MAO B inhibition by **1** and **2** to provide a comparative evaluation. Recombinant human MAO B was expressed in *Pichia pastoris* and purified as detergent-solubilized samples.^27^ We performed steady-state kinetic measurements to determine the *K*_i_ values of these compounds by using the time-course spectrophotometric horseradish-peroxidase-coupled assay and benzylamine as substrate. Initial velocity values measured at different substrate and inhibitor concentrations were fitted to the Michaelis–Menten equation by the program GraphPad Prism 5. For **1**, (±)-**2**, (−)-**2**, and (+)-**2** the best fit (i.e., yielding a *R*^2^ value proximal to 1.0) was obtained with a competitive inhibition model. The *K*_i_ values of (±)-**2**, (−)-**2**, and (+)-**2** were all in the sub-micromolar range with (+)-**2** giving the lowest value (0.093 μM), which is in agreement with the previously determined IC_50_ values (Table 1). In the case of inhibitor **1**, it was observed that each assay measurement (absorbance as a function of time) featured a profile displaying a short window of linearity and reaching a plateau in few minutes, which was uncommon for MAO B and was not detected for compounds **2** (racemate and enantiomers). To probe whether this effect might be related to a tight-binding mode of inhibition previously observed with chromone inhibitors,^28^ we measured IC_50_ values at different enzyme concentrations (at 0.333 mM benzylamine substrate concentration) following well-established protocols^29^ and using the same spectrophotometric assay employed for *K*_i_ determination. Indeed, a linear increase of IC_50_ values was observed (Figure 2A) indicating that **1** is a competitive tight-binding inhibitor of MAO B. An analogous experiment carried out with (±)-**2** revealed that IC_50_ values are not significantly influenced by enzyme concentration, suggesting that this compound is a purely competitive inhibitor (data not shown). Next, to determine an accurate tight-binding *K*_i_ value for **1**, we plotted the ratio between *v*_i_ and *v*_0_ initial enzyme velocities as a function of the inhibitor concentration (Figure 2B). We fitted the data to the Morrison equation^29^ (eq 1):
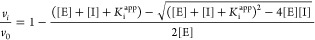
1where *v*_i_ and *v*_0_ are the initial velocity with and without inhibitor **1**, respectively, whereas [E] is enzyme concentration and [I] is inhibitor concentration. The term *K*_*i*_^app^ is the apparent inhibition constant that was used to determine the real inhibition constant (*K*_i_) for competitive tight-binding of inhibitor **1** (4.5 μM, Table 1) by using eq 2:
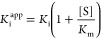
2

**Figure 2:**
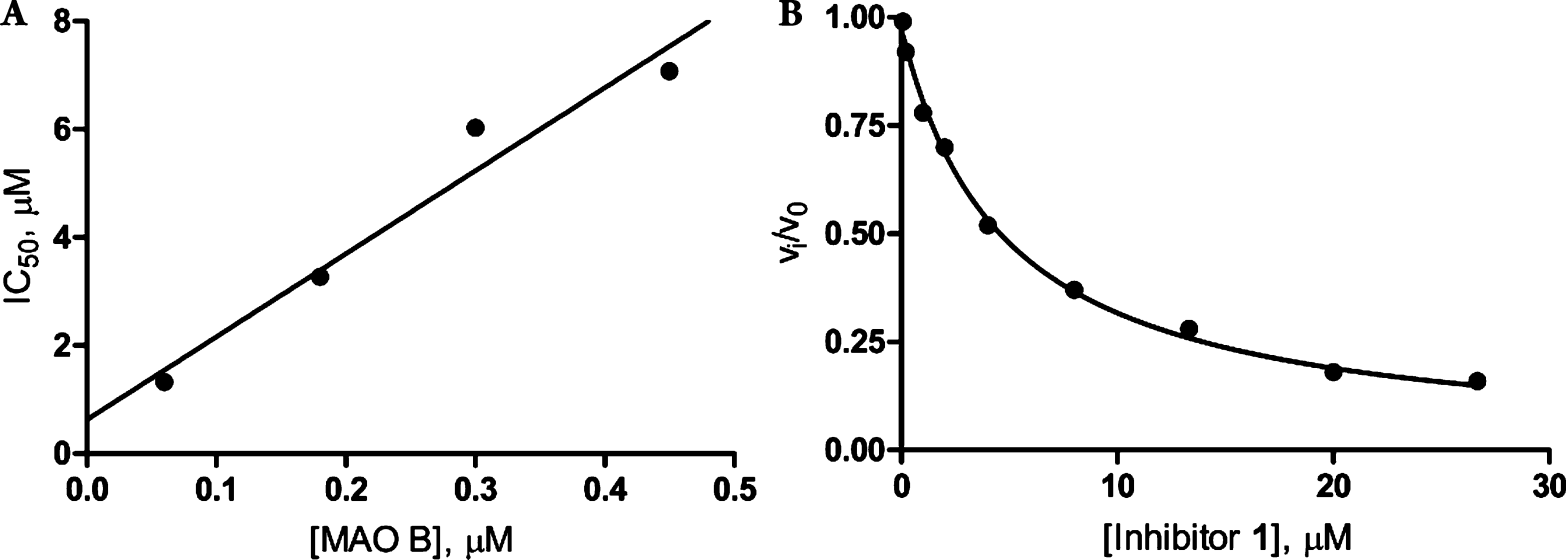
Inhibition of human MAO B by inhibitor **1**. (A) Linear increase of IC_50_ values determined at different enzyme concentrations, indicating a tight-binding mode of inhibition. (B) Plot of enzyme velocity (*v*_i_ and *v*_0_ are the initial velocity values in presence and absence of inhibitor **1**, respectively) as a function of inhibitor concentration. The data points were fitted to the Morrison equation (see text).

The tight-binding *K*_*i*_ for inhibitor **1** is one order of magnitude higher than that initially obtained by fitting the data to the Michaelis–Menten equation for competitive inhibition (0.77 μM). As many tight-binding inhibitors are known to feature a slow onset of inhibition that may be significantly affected by the presence of the enzyme substrate, we investigated the binding mode of these dual-target ligands by biophysical approaches. We carried out a thermal-shift assay to probe the thermostability of MAO B in the presence of the inhibitors compared to the free enzyme by using a TychoTMNT.6 system (NanoTemper Technologies GmbH). The measurements follow the unfolding process of proteins by detecting the intrinsic fluorescence of aromatic residues, rather than using dyes that proved unsuccessful for membrane proteins including MAO B. By using this approach, we determined the melting temperature (*T*_m_) values, which are 56.7 ± 0.2 °C for the free enzyme and 62.2 ± 0.1, 57.7 ± 0.1, and 58.6 ± 0.1 °C for inhibitors **1**, (+)-**2**, and (−)-**2**, respectively (Figure 3). Therefore, all ligands had a stabilizing effect by increasing the enzyme *T*_m_ but **1** determined a remarkable +5.5 °C shift, which was in agreement with its tight-binding inhibition mechanism.

**Figure 3:**
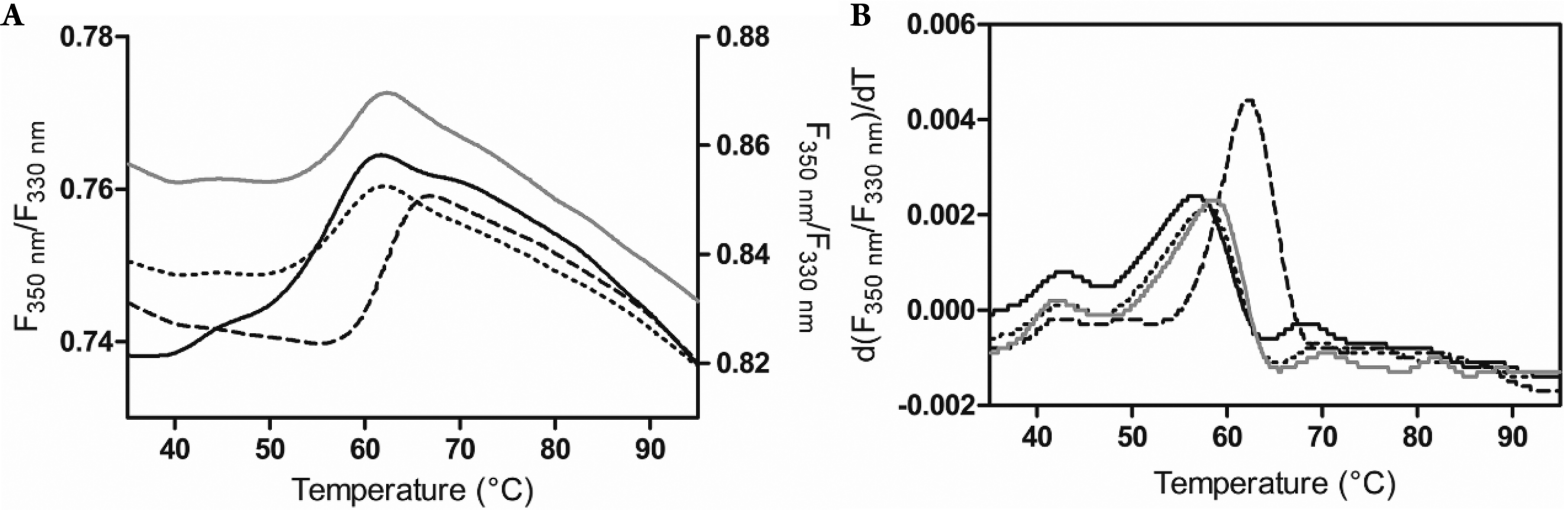
Thermal shift assays to probe the effect of **1**, (+)-**2**, and (−)-**2** inhibitors on human MAO B. The ratio between fluorescence at 350 and 330 nm (detecting Trp and Tyr residues, respectively, getting exposed upon protein unfolding) is plotted as a function of increasing temperature. Continuous black line indicates free MAO B enzyme (values on the left axis), whereas the dashed, dotted, and continuous gray lines correspond to samples of MAO B in the presence of **1**, (+)-**2**, and (−)-**2**, respectively (values on the right axis). (A) Thermal stability curves plotted as normalized fluorescence signal. (B) Graphs displaying the first derivative of the sigmoidal curves showed in panel A, whose peak maximum numbers correspond to the unfolding temperature values (*T*_m_).

Crystallographic studies were carried out to unravel the binding mode of the dual-target inhibitors to human MAO B. The structures of MAO B in complex with **1** and (+)-**2** were solved at 2.3 and 2.1 Å resolution, respectively (Figure 4 and Table 2). The (+)-**2** enantiomer was selected because it displayed a slightly lower *K*_i_ with respect to the (−) analogue and the racemic mixture. No significant differences between chain A and chain B present in the asymmetric unit of either structures were observed (rmsd values for the C_α_ atoms between the two chains are 0.26 and 0.28 Å for the structures in complex with **1** and (+)-**2**, respectively). In the case of the MAO B-(+)-**2** complex, the electron density of the inhibitor piperidine ring was slightly better in monomer B with respect to A although the general conformation of the inhibitor bound to the enzyme active site was the same. We will henceforth refer to monomer A for the description of the structures.

**Figure 4:**
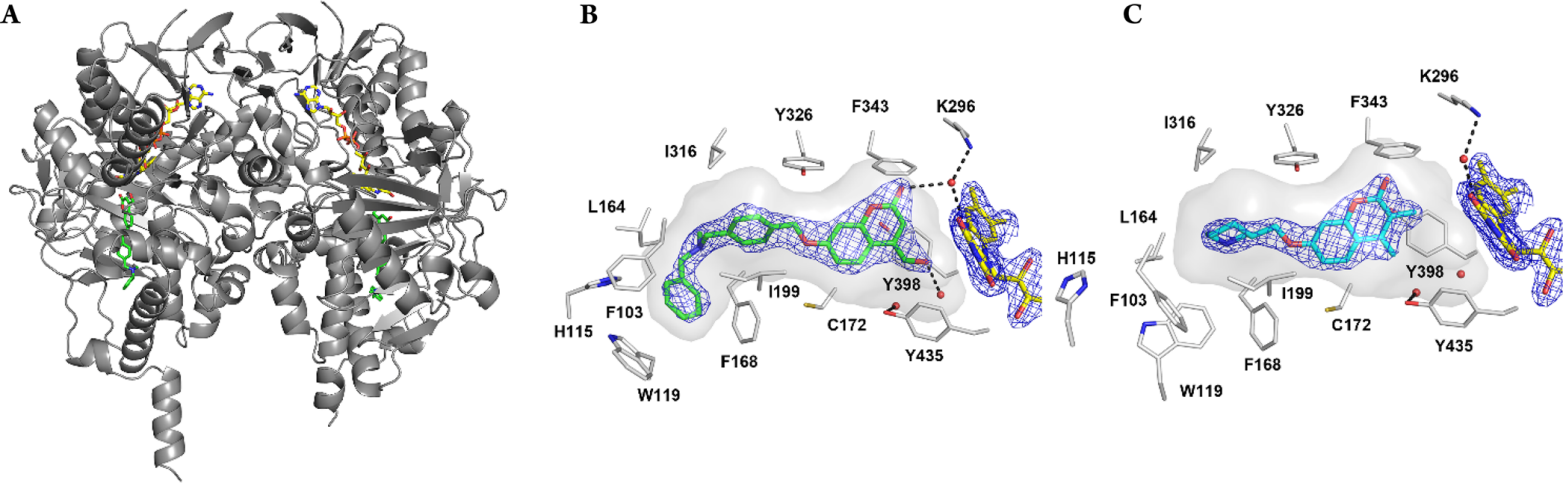
Crystal structures of human MAO B in complex with the dual-target inhibitors. (A) Ribbon diagram of the MAO B overall dimeric structure (in gray) showing inhibitor **1** (in stick style, with carbon, oxygen, and nitrogen atoms in green, red, and blue, respectively) bound within the substrate-binding domain. The FAD cofactor is represented as sticks with carbon atoms in yellow. Chain A is the monomer on the left. (B) Close-up view of the MAO B active site in complex with **1**. The orientation of the molecule is rotated about 90° around a perpendicular axis with respect to Figure 4A (monomer A). Color code is as in Figure 4A, with active site residues drawn with carbon atoms in gray. Water molecules are depicted as red spheres and hydrogen bonds are indicated as dashed lines. The enzyme hydrophobic cavity is represented as gray semitransparent surface). The refined 2F_O_–F_C_ electron density for **1** and FAD (contoured at 1.2 σ) is shown in blue chickenwire mode. (C) Close-up view of the MAO B active site in complex with (+)-**2** bound in the enzyme cavity (carbon atoms in cyan). The protein overall fold (not shown) is essentially identical to that of the structure in complex with **1** displayed in Figure 4A. The aromatic ring of the *N*-benzylpiperidine moiety lacks electron density and was omitted from the structure. All figures of crystal structures were produced with Pymol.^30^

**Table 2:** Data Collection and Refinement Statistics for the Target Enzymes Crystal Structures in Complex with **1** and (+)-**2**

	human MAO B		mouse AChE	
	**1**	(+)-**2**	**1**	(+)-**2**
space group	*C*222	*C*222	*P*2_1_2_1_2_1_	*P*2_1_2_1_2_1_
unit cell axes (Å)	*a* = 131.2	*a* = 131.8	*a* = 78.5	*a* = 79.4
	*b* = 222.4	*b* = 222.7	*b* = 112.7	*b* = 111.0
	*c* = 86.2	*c* = 86.2	*c* = 226.7	*c* = 227.1
resolution (Å)	2.3	2.1	2.6	2.5
PDB code	7P4F	7P4H	7QAK	7QB4
*R*_merge_ (%)^a^^,^^b^	13.4 (68.9)	14.5 (72.0)	7.39 (58.5)	27.4 (163)
C*C*_1/2_ (%)	99.1 (76.7)	98.9 (58.7)	99.7 (95.7)	89.1/67.6
completeness (%)^b^	99.7 (99.9)	98.1 (98.7)	99.6 (99.4)	99.2 (98.1)
unique reflections	56,202	72,527	62,618	70,307
redundancy	5.3 (5.4)	4.4 (4.5)	6.7 (6.9)	6.7 (6.9)
*I*/σ^b^	9.3 (3.7)	6.2 (1.9)	20.1 (4.0)	9.1 (1.3)
no. of non-hydrogen atoms				
protein	7916	7916	8390	8398
inhibitor/cofactor	2x31/2x53	2x21/2x53	2x31/–	2x14/–
detergent^c^	26	26		
water	383	506	129	123
avg B value for protein/inhibitor atoms (Å^2^)	28.9/30.3	27.0/48.6	66.0/68.9	67.9/94.0
*R*_cryst_ (%)^b^^,^^d^	16.1 (20.5)	16.7 (23.8)	19.0 (25.5)	18.4 (26.3)
*R*_free_ (%)^b^^,^^d^	21.3 (27.9)	21.2 (26.7)	22.4 (31.0)^e^	21.7 (28.9)^e^
rms bond length (Å)	0.010	0.009	0.003	0.005
rms bond angles (deg)	1.45	1.59	0.70	0.73

a *R*_sym_ = Σ|*I*_*i*_ – ⟨*I*⟩|/Σ*I*_i_, where *I*_*i*_ is the intensity of the *i*th observation and ⟨*I*⟩ is the mean intensity of the reflection.

b Values in parentheses are for reflections in the highest resolution shell.

c As in previous human MAO-B structures, one molecule of the Zwittergent 3–12 detergent (used in crystallization experiments) is partly visible in the electron density of each of the two protein monomers present in the asymmetric unit.

d *R*_cryst_ = Σ|*F*_obs_ – *F*_calc_|/Σ|*F*_obs_|, where *F*_obs_ and *F*_calc_ are the observed and calculated structure factor amplitudes, respectively. *R*_cryst_ and *R*_free_ were calculated using the working and test (randomly chosen reflections) sets, respectively.

The electron density map of both structures was of good quality and allowed us to unambiguously identify the position of inhibitors **1** and (+)-**2** in the hydrophobic cavity of MAO B (Figure 4B and C). The coumarin moiety is bound in front of the flavin ring with the coumarin lactone-group pointing upward in proximity of Phe343 and Lys296. In the structure of MAO B in complex with **1**, the hydroxymethyl substituent at C4 lies between the Tyr398-Tyr435 pair forming the “aromatic sandwich” found in other flavin-dependent amine oxidases.^31^ Instead, (+)-**2** binds slightly more distant from the flavin, in the exact position found for the coumarin inhibitors previously studied by our group (Figure 5). This is likely due to the length of **1** and its peculiar binding mode to MAO B active site. The bis-*N*-benzylamine moiety is wedged into a pocket of the entrance cavity adopting a hook conformation that was never observed in other MAO B structures (Figure 4B). This binding mode induce significant structural adjustments of the residues lining this portion of the active site (Figure 5). Phe103 and Trp119, which belong to the surface loop giving access to the enzyme cavity, are displaced by the terminal aromatic ring of **1**, and, in turn, the side chains of His115 and Leu164 adopt a different conformation. As a result, the shape of the cavity, whose plasticity has been so far limited to the open/closed switch of Ile199 leading to the bipartite architecture of the MAO B active site,^32^ is in this case conserved in proximity of the flavin but significantly transformed nearby the protein surface (Figure 4B). Compound (+)-**2** binds similarly to the previously studied coumarin inhibitors (Figure 5). Unfortunately, no clear electron density was observed for the terminal aromatic ring of (+)-**2** that, together with the piperidine ring, forms the donepezil-inspired moiety targeting AChE. The possibility that the inhibitor was previously oxidized by the enzyme at the tertiary amino group was ruled out by high resolution mass analysis (Q-TOF, data not shown), which suggests that this part of the inhibitor molecule is disordered. In addition, the exact configuration of the chiral center within the piperidine ring could not be determined because the electron density is compatible with either *R* or *S*. We tentatively modeled the inhibitor in the *R* configuration because it corresponds to the best fit with the ring in chair conformation.

**Figure 5:**
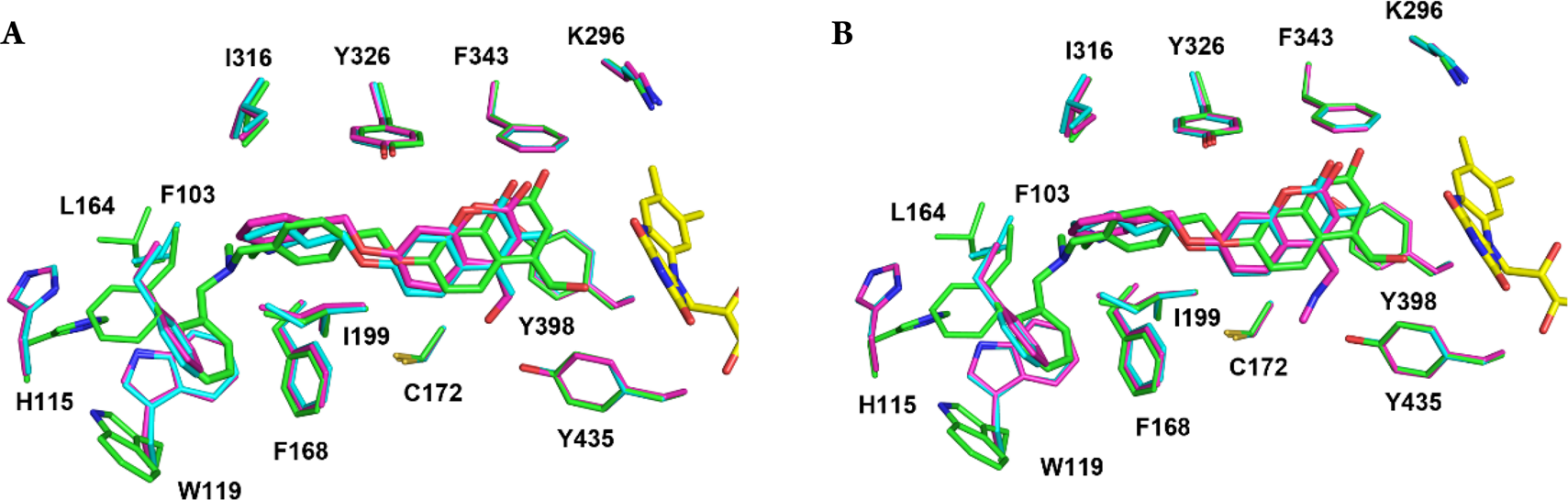
Structural comparison of **1** and (+)-**2** with other coumarin inhibitors previously studied.^26^ Superposition of the MAO B active site structures in complex with **1** (green) and (+)-**2** (cyan) onto that in complex with (A) 7-(3- chlorobenzyloxy)-4-carboxaldehyde-coumarin (PDB code 2V60) and (B) 7-(3- chlorobenzyloxy)-4-(methylamino)methyl-coumarin (PDB code 2V61), drawn in magenta in their respective panels. Active site residues are colored accordingly to highlight structural differences (oxygen and nitrogen atoms are in red and blue, respectively, as in the previous figures). For the sake of clarity, water molecules were removed.

The IC_50_ values for **1**, (+)-**2**, and (−)-**2**, which were also measured on purified mouse AChE (Table 1), are in agreement with those obtained with the human enzyme, validating the mouse enzyme as a proper model for this dual-target analysis. The structures of mouse AChE (hereafter indicated simply as AChE) were determined to a resolution of 2.6 and 2.5 Å for the complex with **1** and (+)-**2**, respectively (Table 2). The asymmetric unit contains two copies of AChE with similar quality of the electron density map, and we will refer to monomer A for description of the structure (Figure 6A). The electron density maps clearly defined the entire binding mode of **1** (Figure 6B), whereas in the case of (+)-**2** only the coumarin moiety could be modeled (Figure 6C). Nevertheless, the structures unambiguously show that **1** and (+)-**2** have two distinct binding modes (Figure 6D). In particular, as shown in Figure 6A, **1** behaves as a dual-binding site inhibitor occupying both the PAS and the CAS. This is relevant because **1** combines the blockade of ACh degradation with the occupancy of PAS whose chaperone-like activity accelerates β-amyloid deposition into oligomers.^14^ The structure of **1** in complex with AChE shows nearly parallel arene–arene interactions involving the coumarin moiety of **1** in a sandwich-like binding with both the phenol ring of Tyr124 and the indole ring of Trp286. The coumarin C=O group may form a hydrogen bond to the main chain nitrogen of Ser298 (O···N distance of 2.8 Å). The binding of the coumarin group is facilitated by a significant structural change of Trp286, similar to previously reported for other ligands such as the oxime HI-6.^33^ The *para*-xylyl moiety of **1** is in contact distance to the hydroxyl group of Tyr124 and also forms π–π stacking interactions with the aromatic rings of Phe338 and Tyr341. The phenol ring of Tyr337 is slightly shifted and rotated to accommodate the tertiary amino group of **1**, whereas the terminal aromatic ring forms parallel arene–arene interactions with the indole of Trp86.

**Figure 6:**
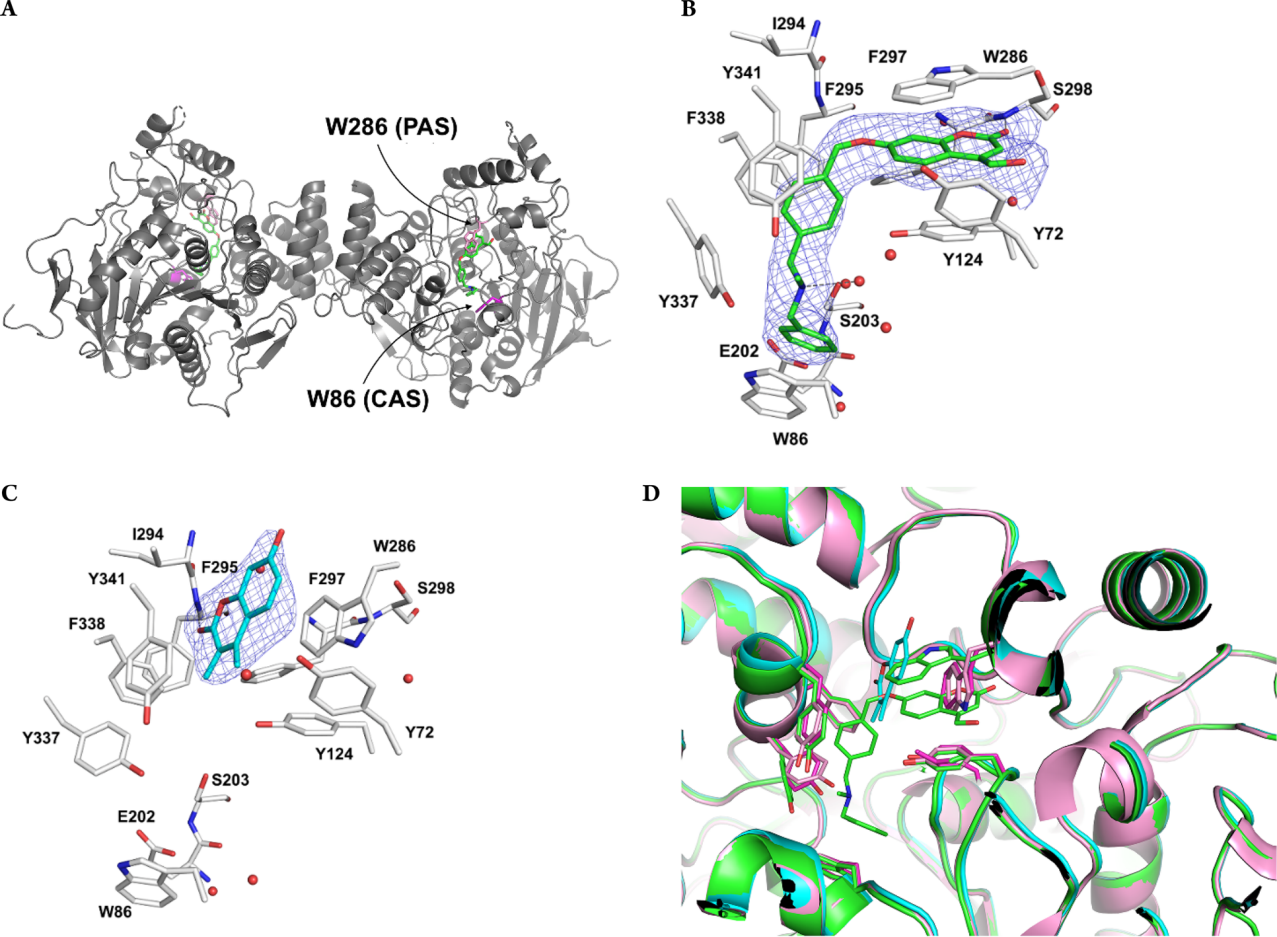
Crystal structures of mouse AChE in complex with **1** and (+)-**2**. (A) Ribbon diagram of the AChE dimer (in gray) showing the binding of inhibitor **1** (green) in proximity to the CAS and the PAS of the enzyme. Atom color code is as in Figure 4. Zoomed view of the binding of **1** in green (B) and (+)-**2** in cyan (C) with potential hydrogen bonds depicted as dashed lines and the Fo–Fc simulated annealing electron density omit map shown at a contour level of 3σ shown in blue. (D) Superposition of the structures of AChE in complex with **1** (green) and (+)-**2** (cyan) and the free enzyme (i.e., with no inhibitor bound, PDB code 1J06, in magenta). For the sake of clarity, residue labels were omitted.

In contrast to the deeply buried binding pose of **1**, the structure of the complex between (+)-**2** and AChE shows a shallow mode of binding with the coumarin ring system forming parallel displaced arene–arene interactions with the indole of Trp286 and Tyr341 side chain (Figure 6C and D). The lactone carbonyl of (+)-**2** is found at hydrogen bonding distance to the main chain nitrogen of Phe295 (O···N distance of 3.0 Å). The conformation of Trp286 is nearly identical to the conformation found in the apo structure of AChE (PDB code 1J06). The linker, the piperidine ring, and the terminal benzylic moiety of (+)-**2** are directed toward the bulk solvent, not defined by the electron density map and consequently not modeled in the final structure.

In summary, the present study provides a structural template for the rational design of dual AChE-MAO B reversible inhibitors containing a coumarin moiety. Compounds (±)-**2**, (−)-**2**, and (+)-**2** showed MAO B binding affinity similar to that of the previously studied coumarin analogues^26^ (*K*_i_ in the 0.1–0.4 μM range), and also the interaction with the enzyme active site is well conserved (Figure 5). In particular, the coumarin ring is lodged in close contact to the flavin in a similar fashion, with the main difference residing in the donepezil-inspired basic chain that is partly disordered and likely sprouting out of the cavity (Figure 4C). This partially defined binding mode is even more pronounced in AChE with (+)-**2** bound with the coumarin ring near the surface of the protein without any significant conformational change in the surrounding residues with respect to the free enzyme (Figure 6C). Instead, compound **1** is fully visible and shows a better fitting within the binding pockets of both MAO B and AChE, but at the expense of significant conformational changes in the active site of the enzymes. In MAO B, the coumarin moiety and the linker of **1** are forced to stay longitudinal by the rigid enzyme cavity and the terminal aromatic ring is accommodated in a niche of the entrance cavity rather than protruding out of the protein surface as observed with (+)-**2** (Figure 4A). This unusual binding is allowed by conformational changes of the residues in the rear of the cavity (His115, Trp119, Phe103; Figure 5A). Likewise, in AChE, inhibitor **1** is fully buried in the protein structure, adopting a U-shaped conformation allowed by the structural change of Trp286 and Tyr337 side chains (Figure 6B). The binding affinity of **1** to AChE is indeed 1 order of magnitude better than that of (+)-**2** (Table 1), whereas in the case of MAO B the two compounds display similar IC_50_ values but compound **1** shows a tight-binding mode of inhibition. In conclusion, this study provides the basis to develop dual-target MAO B/AChE inhibitors based on the coumarin/donepezil scaffolds with different structural details that may be used to modulate the binding affinity to the enzymes.

## Supporting Information Available

Experimental details and protocols on protein expression and purification, inhibition and binding studies, X-ray crystallography (PDF)

## Abbreviations

AD: Alzheimer’s disease
PD: Parkinson’s disease
MTDL: multitarget directed ligands
MAO: monoamine oxidase
AChE: acetylcholinesterase
CAS: catalytic anionic subsite
PAS: peripheral anionic subsite
