# Supplementary material for: Dual Reversible Coumarin Inhibitors Mutually Bound to Monoamine Oxidase B and Acetylcholinesterase Crystal Structures
Source: ACS Med Chem Lett. 2022 Feb 18;13(3):499–506. doi: 10.1021/acsmedchemlett.2c00001 (PMC8919507; doi:10.1021/acsmedchemlett.2c00001)
Supplement: Supplementary file 1 — ml2c00001_si_001.pdf [file ml2c00001_si_001.pdf]

SUPPORTING INFORMATION

Dual reversible coumarin inhibitors mutually bound to Monoamine Oxidase B and Acetylcholinesterase crystal structures

Fredrik Ekström[¶,§], Andrea Gottinger[†,§], Nina Forsgren[¶], Marco Catto[‡], Luca G. Iacovino[†,ǁ], Leonardo Pisani[‡]*, Claudia Binda[†]*

¶Swedish Defence Research Agency, CBRN Defence and Security, Umeå, Sweden

†Dept. of Biology and Biotechnology, University of Pavia, 27100 Pavia, Italy

‡Dept. of Pharmacy-Pharmaceutical Sciences, University of Bari "Aldo Moro", via E. Orabona 4, 70125, Bari, Italy

*Co-corresponding authors: Claudia Binda: claudia.binda@unipv.it, phone +39-0382-985527; Leonardo Pisani: leonardo.pisani@uniba.it, Phone +39 080-5442803

§These authors contributed equally to this work

ǁPresent address: FoRx Therapeutics AG, c/o Novartis Pharma AG, WSJ-350.3.04, Lichtstrasse 35, 4056 Basel, Switzerland

Contents

Experimental section

1. Reagents S2

2. Expression and Purification of recombinant proteins and enzymatic activity assays S2

3. Inhibition studies S2

4. Thermal shift analysis on recombinant human MAO B S3

5. X-ray crystallography S4

6. References S5

Experimental section

1. Reagents. All reagents were purchased from Sigma-Aldrich, except for detergents that were provided by Anatrace (USA).

2. Expression and Purification of recombinant proteins and enzymatic activity assays. Human recombinant MAO B was expressed in *Pichia pastoris* and purified following published protocols.[1] Purified protein samples in 50 mM potassium phosphate buffer pH 7.5, 0.8% (w/v) β-octylglucoside, 20% (w/v) glycerol was concentrated by Amicon30K (Millipore) up to about 50 μM, determined by measuring the absorbance at the flavin peak (ε_{456} = 12,000 M^{-1} cm^{-1}) using a NanoDrop ND-1000 spectrophotometer (Thermo Scientific). The MAO B enzymatic activity was measured in a 100 μL cuvette by the peroxidase-coupled assay[2] at 25 °C using benzylamine as substrate. The reaction was started by adding 0.1 μM enzyme (final concentration) in 50 mM Hepes/NaOH pH 7.5, 0.25% (v/v) reduced Triton X-100, 0.1 M 4-aminoantipyrine, 1 mM 3,5-dichloro-2-hydroxybenzenesulfonic acid, 0.01 mg ml^{-1} horseradish peroxidase. Increase of Abs at 515 nm (ε_{515} = 26,000 M^{-1} cm^{-1}) as a function of time was monitored using a Cary100 spectrophotometer (Agilent Technologies, CA, USA). All experiments were performed in duplicate.

Mouse AChE was expressed in HEK293 F cells and purified as previously reported.[3] The activity of AChE was measured using the Ellman assay adaped to a 96-well plate format.[4] The concentration of 5,5′-dithiobis(2-nitrobenzoic acid) was 0.2 mM and the concentration of acetylthiocholine iodide was 1 mM. The measurements were performed in 0.1 M phosphate buffer set at a pH of 7.4 in a final assay volume of 200 μL using a Infinite M200 plate reader (Tecan).

3. Inhibition studies. Determination of K_i values was performed through steady-state kinetic analysis by measuring the initial velocities v_0 (peroxidase-coupled assay) of substrate oxidation in the presence of varied inhibitor concentration and fitting the data to the Michaelis-Menten equation (using GraphPad Prism software 5.0). The tight-binding inhibition mechanism of the inhibitors was tested by measuring the IC_{50} values using the same assay at fixed substrate concentration (0.333 mM benzylamine) and increasing enzyme concentration (Figure 2A in the main text). IC_{50} values were obtained by fitting percentage of inhibition versus log(inhibitor concentration) using GraphPad software 5.0. Tight-binding K_i value for **1** was determined through a dose-response curve by plotting the enzyme activity (ratio between v_i and v_0, i.e. initial velocities with and without inhibitor, respectively) at a fixed substrate concentration (2.7 mM benzylamine) and at varying concentrations of **1**. Data were fitted using the Morrison equation[5] in GraphPad Prism software 5.0. Determination of IC_{50} values for the compounds inhibition of AChE was performed as previously reported.[6] At least 5 replicate IC_{50} determinations at 8 inhibitor concentrations were performed for each compound. The obtained dose-response curve was fitted using the response variable slope versus log(inhibitor concentration) equation (four parameters) in GraphPad Prism software 5.0.

4. Thermal shift analysis on recombinant human MAO B. Thermostability assays were performed to evaluate the effect of the inhibitors on the stability of the protein using a TychoTMNT.6 system (NanoTemper Technologies GmbH, Munich, Germany). The experiments were carried out in triplicate, in the presence and absence of inhibitors. The protein concentration was adjusted to 1 mg/mL by diluting with the purification buffer (50 mM potassium phosphate buffer pH 7.5, 0.8% (w/v) β-octylglucoside, 20% /w/v) glycerol) and incubated for fifteen minutes with a 5-fold molar excess of inhibitor. The ratio between fluorescence at 350 nm and at 330 nm, tryptophan and tyrosine respectively, was measured heating up from 35 °C to 95 °C for the duration of three minutes and derived to determine the inflection point corresponding to T_m.

5. X-ray crystallography. Human MAO B was gel filtered in 25 mM potassium phosphate pH 7.5, 8.5 mM Zwittergent 3-12 and co-crystallized with **1** and **(+)-2** by the sitting-drop vapour diffusion method following published protocols.[7] X-ray diffraction data were collected at the beamlines of the Swiss Light Source in Villigen (Switzerland) and European Synchrotron Radiation Facility in Grenoble (France). Crystals were soaked into a mother liquor solution containing 18% (v/v) glycerol and flash-cooled in a stream of gaseous nitrogen at 100 K. Data processing and scaling (Table 2) were performed using XDS[8] and the CCP4 package.[9] The coordinates of MAO B in complex with safinamide (PDB code 2V5Z), after removal of all water and inhibitor atoms, were used as initial model. The programs Coot[10] and REFMAC5[11] were used for model building and refinement. Figures were generated by the program Pymol.[12]

AChE was crystallized as previously reported.[3] The crystals were soaked with several 1 μL portions of **1** or **(+)-2** dissolved in 30 % (v/v) polyethylene glycol 750 monomethylether, 100 mM HEPES pH 7.1. The crystals were subsequently incubated for 70 hours prior to vitrification and data collection at the MAXIV BioMAX beamline in Lund (Sweden) equipped with an Eiger 16M Hybrid-pixel detector (DECTRIS. Switzerland). Data processing and scaling were performed using XDS[8] and the CCP4 package.[9] The coordinates of apo AChE (PDB code 1J06) were used as a model and the initial structure was determined using difference Fourier methods using the Phenix program suite.[13] The atomic coordinates were modelled according to the $2|Fo| - |Fc|$ and $|Fo| - |Fc|$ maps using the program Coot.[10] We note that for unknown reasons, **1** has significantly better merging- and scaling statitics than **(+)-2**. Furthermore, the electron density maps of **(+)-2** show an unknown ligand binding in the vicinity of Ser203Oγ. Angle and bond restraints of the ligand were optimized by the phenix.elbow option using the eLBOW AM1 algorithm. The electron density maps shown in Figure 6 (main text) were calculated following simulated annealing refinement using a coordinate file from which coordinates from **1** or **(+)-**

2 were removed. The molecular graphics and figures were produced using the PyMOL Molecular Graphics System, Version 2.3.4.[12]

6. References

(1) Newton-Vinson, P.; Hubalek, F.; Edmondson, D. E. High-Level Expression of Human Liver Monoamine Oxidase B in Pichia Pastoris. *Protein Expr. Purif.* **2000**.

(2) Vojinović, V.; Azevedo, A. .; Martins, V. C. .; Cabral, J. M. .; Gibson, T. .; Fonseca, L. . Assay of H2O2 by HRP Catalysed Co-Oxidation of Phenol-4-Sulphonic Acid and 4-Aminoantipyrine: Characterisation and Optimisation. *J. Mol. Catal. B Enzym.* **2004**, *28* (2–3), 129–135.

(3) Ekström, F.; Akfur, C.; Tunemalm, A.-K.; Lundberg, S. Structural Changes of Phenylalanine 338 and Histidine 447 Revealed by the Crystal Structures of Tabun-Inhibited Murine Acetylcholinesterase. *Biochemistry* **2006**, *45* (1), 74–81.

(4) Ellman, G. L.; Courtney, K. D.; Andres, V.; Featherstone, R. M. A New and Rapid Colorimetric Determination of Acetylcholinesterase Activity. *Biochem. Pharmacol.* **1961**, *7* (2), 88–90.

(5) Henderson, P. J. A Linear Equation That Describes the Steady-State Kinetics of Enzymes and Subcellular Particles Interacting with Tightly Bound Inhibitors. *Biochem. J.* **1972**.

(6) Berg, L.; Andersson, C. D.; Artursson, E.; Hörnberg, A.; Tunemalm, A.-K.; Linusson, A.; Ekström, F. Targeting Acetylcholinesterase: Identification of Chemical Leads by High Throughput Screening, Structure Determination and Molecular Modeling. *PLoS One* **2011**, *6* (11), e26039.

(7) Binda, C.; Wang, J.; Pisani, L.; Caccia, C.; Carotti, A.; Salvati, P.; Edmondson, D. E.; Mattevi, A. Structures of Human Monoamine Oxidase B Complexes with Selective Noncovalent Inhibitors: Safinamide and Coumarin Analogs. *J. Med. Chem.* **2007**, *50* (23), 5848–5852.

(8) Kabsch, W. XDS. *Acta Crystallogr. Sect. D Biol. Crystallogr.* **2010**, *66* (2), 125–132.

(9) Collaborative Computational Project, N. 4. The CCP4 Suite: Programs for Protein Crystallography. *Acta Crystallogr. Sect. D Biol. Crystallogr.* **1994**, *50* (5), 760–763.

(10) Emsley, P.; Lohkamp, B.; Scott, W. G.; Cowtan, K. Features and Development of Coot. *Acta Crystallogr. Sect. D Biol. Crystallogr.* **2010**, *66* (4), 486–501.

(11) Murshudov, G. N.; Skubák, P.; Lebedev, A. A.; Pannu, N. S.; Steiner, R. A.; Nicholls, R. A.; Winn, M. D.; Long, F.; Vagin, A. A. REFMAC 5 for the Refinement of Macromolecular Crystal Structures. *Acta Crystallogr. Sect. D Biol. Crystallogr.* **2011**, *67* (4), 355–367.

(12) DeLano, W. L. Use of PYMOL as a Communications Tool for Molecular Science. In *Abstr. Pap. Am. Chem. Soc.*; 2004; pp 228, U313–U314.

(13) Liebschner, D.; Afonine, P. V.; Baker, M. L.; Bunkóczi, G.; Chen, V. B.; Croll, T. I.; Hintze, B.; Hung, L.-W.; Jain, S.; McCoy, A. J.; Moriarty, N. W.; Oeffner, R. D.; Poon, B. K.; Prisant, M. G.; Read, R. J.; Richardson, J. S.; Richardson, D. C.; Sammito, M. D.; Sobolev, O. V.; Stockwell, D. H.; Terwilliger, T. C.; Urzhumtsev, A. G.; Videau, L. L.; Williams, C. J.; Adams, P. D. Macromolecular Structure Determination Using X-Rays, Neutrons and Electrons: Recent Developments in Phenix. *Acta Crystallogr. Sect. D Struct. Biol.* **2019**, *75* (10), 861–877.
